# The personal value of being part of a Tropical Health Education Trust (THET) links programme to develop a palliative care degree programme in Sub Saharan Africa: a descriptive study of the views of volunteer UK health care professionals

**DOI:** 10.1186/s12992-015-0136-6

**Published:** 2015-12-14

**Authors:** B. A. Jack, J. A. Kirton, J. Downing, K. Frame

**Affiliations:** Evidence-Based Practice Research Centre, Edge Hill University, St Helens Road, Ormskirk Lancs, UK; Imperial College Hospital NHS Trust, Paddington, London, W2 1NY UK; Makerere University, PO Box 7062, Kampala, Uganda

**Keywords:** Palliative care, End of life, Cancer, Volunteering

## Abstract

**Background:**

There is a global need to expand palliative care services to reach the increasing number requiring end of life care. In developing countries where the incidences of cancer are rising there is an urgent need to develop the palliative care workforce. This paper reports on a UK Department for international development (DFID) initiative funded through the Tropical Health Education Trust (THET) where palliative care staff, both clinical and academic, volunteered to help to develop, support and deliver a degree in palliative care in sub-Saharan Africa. The objective of the study was to explore the personal impact on the health care professionals of being part of this initiative.

**Methods:**

An evaluation approach using a confidential electronic survey containing quantitative and qualitative questions was distributed to all 17 volunteers on the programme, three months after completion of the first cohort. Data were analysed using descriptive statistics and content thematic analysis. Ethical review deemed the study to be service evaluation.

**Results:**

82 % (14) responded and several themes emerged from the data including the positive impact on teaching and educational skills; clinical practice and finally personal development. Using a score of 1–10 (1-no impact, 10 maximum impact) ‘*Lifestyle choices - life work balance’* (rating 7.83) had the most impact.

**Conclusions:**

This approach to supporting the development of palliative care in Sub-Saharan Africa through skill sharing in supporting the delivery of a degree programme in palliative care was successful in terms of delivery of the degree programme, material development and mentorship of local staff. Additionally, this study shows it provided a range of positive impacts on the volunteer health care professionals from the UK. Professional impacts including increased management skills, and being better prepared to undertake a senior role. However it is the personal impact including lifestyle choices which the volunteers reported as the highest impact. Interestingly, several of the faculty have joined other volunteer programmes to continue to support the international development of palliative care.

## Background

### The need for education of the palliative care workforce in Sub Saharan Africa

The need for palliative care around the world is immense and there is a specific need for service leaders, clinical palliative care providers and trained trainers for palliative care. Non communicable diseases, including cancer, are the leading cause of death around the world [1], with 8.2 million cancer deaths in 2012 (38 % more than 20 years ago [2], and an estimated 14.1 million new cancer cases [3, 4]. The rates of cancer are expected to increase by over 400 % over the next 50 years (due to the increasing total population, increasing average age and the broad epidemiological transitions with a shift towards NCDs and multiple morbidity) with 70 % of new cancer cases being in the developing world [2]. One of the challenges to providing cancer care in Sub-Saharan Africa is late presentation, approximately 80 % of cancer patients present with advanced, and incurable disease. Limited availability of diagnostic and treatment facilities impacts on the prognosis of those presenting early, cancer mortality and morbidity are high thus furthering the need for palliative care. Sub-Saharan Africa bears the burden of communicable disease, in particular HIV, with 25 million adults and children living with HIV and 1.2 million deaths per year [5]. The Global Atlas of Palliative Care at the End of Life [6], ( states that in 2011 there were 54.6 million deaths, 66 % of them due to non communicable diseases, and over 29 million dying from conditions requiring palliative care (94 % adults, 69 % > 60 years of age and 6 % <15 years). Therefore 377 per 100,000 adults and 63 children per 100,000 children are estimated to need palliative care at the end of life.

Palliative care service provision in Sub-Saharan Africa has improved since it was first mapped in 2006 [7], however despite areas of significant palliative care development there are huge populations across Africa with no access to palliative care [8]. Knapp et al. [9] undertook a systematic review of the provision of children’s palliative care noting that 65.5 % of countries around the world had no known children’s palliative care provision, 18.8 % had capacity building activities, 9.9 % localised community palliative care provision and only 5.7 % of countries around the world had community palliative care provision reaching mainstream providers. In 2011 the WHO estimated that 80 % of the world’s population have no access to morphine for pain relief [10]. Affordable and effective models of palliative care exist throughout Africa however as illustrated there is an immense need for palliative care in Sub-Saharan Africa and huge gaps in its provision. Therefore education and training is a foundation measure for the ongoing development of palliative care [11], and was the inspiration for the degree in Palliative Care in Sub-Saharan Africa.

Hospice Africa Uganda was founded in 1993 in Kampala, with a vision to initiate and support palliative care throughout Africa. Clinical services have developed as well as parallel education programmes for health and supportive care professionals at undergraduate and postgraduate level. The Bachelor of Science in Palliative Care for Africa degree programme (the focus of this specific study) a joint venture between Hospice Africa Uganda education unit (the Institute of Hospice and Palliative Care in Africa (IHPCA), and Makerere University who award the degree, was launched in 2009.

The degree in Palliative Care for Africa course does not just encompass clinical palliative care training and skill development, but also provides training in needs assessment, models of service delivery, management, donor liaison, writing funding proposals, advocacy research and teaching skills. The degree equips graduates with the necessary skills to return to their places of work to develop services, train others and develop the evidence base required to further develop palliative care in Africa in addition to providing good quality palliative care. To date: 166 have completed the Diploma in PC, 46 the Degree in PC and there are currently 20 students in year 1, 24 in year 2 and 19 in year 3. Participants come from across the region and not just within Uganda, thus developing the workforce in a range of countries including Uganda, Cameroon, Kenya, Tanzania, Nigeria, Rwanda, Zambia, and Zimbabwe.

### The Tropical Health Education Trust (THET) Links Health Partnership

The underpinning goal of THET is to harness the expertise of health care workers in the UK, to work with staff in the lower income countries, to enable them to increase their capacity to provide health care. Central to the goal, is the embracement of international volunteering by UK clinical staff. Within palliative care, volunteering plays a pivotal role with approximately 125,000 volunteers in hospices in the UK alone [12]. Globally the establishment of the specialty of palliative care has seen the concomitant rise in volunteers, particularly in the western world and Australia. With palliative care emerging in resource poor countries, there has seen the emergence of models of volunteer workers, often trained to support the hospice team. Studies reporting the personal impact of volunteering in palliative care settings are emerging including a Canadian study by Claxton – Oldfield who found volunteers reported a change in personal growth [13]. More recently in developing countries including India and Sub Saharan Africa, the volunteers are reporting a growth in personal pride and valuing the roles [14–16].

The THET Health – Links model offers opportunity for a link between a UK NHS organisation and a health facility or organisation in a developing country. Staff with various roles within the UK organisation, can have the opportunity to contribute to supporting capacity building of health care staff in the link organization abroad. Responding to the needs of that organisation. The link model is flexible and may include multiple short trips, as well as longer periods of leave. This study relates to a THET Health links partnership established between a UK virtual palliative care faculty (established specifically for this project) and the education team at Hospice Africa Uganda to support the development and delivery of a palliative care degree over a three year period. The faculty comprised palliative care multi-disciplinary staff (nurses, doctors, social workers, chaplains) and academic staff from across the UK, employed in the National Health Service (NHS), academic institutions and the voluntary sector, for example hospices. The UK faculty supported the Ugandan team, in the development and delivery of the degree programme for 30 health professionals annually from different African countries. UK faculty members were volunteering their time, whilst their accommodation and travel was funded through the UK Shoestring Palliative Care LINKS Programme, the project had a grant from the International Health Links Funding Scheme, which was funded by the UK Department for International Development and the Department of Health, and jointly managed by THET and the British Council. This paper will report on the personal value of being a volunteer, and reports on part of a wider study for the funders, which looked at the impact of the partnership on the volunteers, students and partners [copy available from the corresponding author]

Whilst supporting the degree programme, UK palliative care faculty undertook a range of activities including: supporting the development of the curriculum content (syllabus review), teaching, student support, mentoring Ugandan degree teaching staff, assessment activities including setting examinations and assignment questions as well as marking and moderating and helping with the practical examination assessment. Additionally faculty members also undertook clinical work as appropriate, including home visits, working with the hospital palliative care team and outpatient consultations.

Aim of the Study: As part of the evaluation of the partnership, we aimed to explore the impact on the UK faculty of participating in the partnership. Data collection was between November and December 2012, following completion of the first cohort of the degree programme and analysis completed in 2013.

## Methods

An evaluation approach was selected for the study, which is widely used in both education and health service research [17–19]. Both a quantitative and qualitative focus was included to ensure all relevant data for THET was obtained, as well as a qualitative element, to enable participants to draw upon their experiences, was captured within the study design [20, 21].

A descriptive survey was adopted for the study offering a pragmatic approach as the faculty were based across the UK, as well as providing a degree of confidentiality to encourage people to express honest views and opinions [21, 22]. The survey contained an information section regarding the purpose of the evaluation, assurance of their confidentiality and permission to use written quotes. The return of the survey was considered as them granting consent to participate. Survey Monkey® was used to develop a confidential survey that was distributed electronically. The development of the questions was undertaken by the research team and the questionnaire was purposefully designed not to collect demographic information or any other potentially identifying information due to the small number of participants. It was felt that this would encourage candour, prevent potential identification of individuals from this small population and insure anonymity of research participants. Data were analysed using descriptive statistics via Survey Monkey®^.^ [21, 23].

The open-ended free text questions were subject to content thematic analysis [20, 21]. The survey was sent out to 17 members of the faculty, the original number of the faculty at the commencement of the partnership. However, it must be noted that three members had been inactive in the partnership over the latter year, due to personal commitments and finally responses were received from 14 members (82 % response rate). The respondents current posts comprised; academic, clinical doctor (ranging from specialist trainee to consultant and GP), specialist nurses and chaplains. All but one of the respondents had undertaken visits to Uganda to support the programme and were involved with teaching, clinical work, clinical experience, service review and research.

Ethics: The project was reviewed by West London Research Ethics Committee and considered service evaluation. Respondents are referred to by number against the quotations, due to the small sample size to maintain confidentiality [24]. Respondents were invited to participate in the survey and advised that completion of the survey implied consent for publication.

## Findings

Several themes emerged from the data including the impact on teaching and educational skills; clinical practice and finally personal development.

### Impact of the visit on teaching and educational skills

Seven (50 %) of the respondents noted how the visit had impacted upon their UK practice; examples included teaching, writing degree materials and delivery, including practice based learning. Eight commented it had improved their teaching. Comments included the impact upon how they delivered sessions including:

*“I learnt about Practice Based Learning as a teaching tool and by attending all the presentations within the module I also learnt other teaching techniques used by the other facilitators”* UK faculty respondent 2

*“More interactive methods (the African students were not fans of didactic teaching although did like written information to be provided)”* UK faculty respondent 9

Interestingly the change in teaching style and less reliance on technology was also noted:

*“I am more confident to deliver sessions without PowerPoint and they are generally more interactive.”* UK faculty respondent 3

The availability of the consolidated residential weeks also enabled the UK faculty to gain a lot of teaching practice and one respondent noted this impacted upon their confidence:

*“More confidence because of the amount of teaching I did whilst there.”* UK faculty respondent 10

### Clinical practice

The impact on their clinical skills was also reported including working with limited resources, greater knowledge of symptoms related to HIV/AIDS, increased cultural awareness and increased confidence. Examples were given by several respondents, this included developing cultural awareness particularly in relation to resource issues;

“*Costs effective investigation and management - syndromic management approach, cultural awareness and skills working through interpreters”* UK faculty respondent 12

Similarly the respondents reported an increased awareness of caring for patients with HIV/AIDS;

*“I learnt more about HIV/AIDS and its effects on the individual patient and their family which will help with caring for UK patients”* UK faculty respondent 2

There was also the impact of working without as much supervision as they were used to and one respondent stated:

*“Previously, as a trainee there was always someone else to ask, but I now have more confidence in my abilities and feel ready for a Consultant post.”* UK faculty respondent 10

Interestingly, the experience working with restricted investigations was also seen as valuable:

*“Much more aware of working without access to normal range of investigations and interventions. This is particularly valuable in the field of palliative care so that we remain patient and not disease focused”* UK faculty respondent 8

Only four respondents referred to the impact of the visit on research, with one reporting a joint conference presentation with a member of the Ugandan team.

### Personal development

Eleven respondents commented on the impact of the visit on their own personal development. This covered a variety of areas including; developing a love of travel, increased understanding of African issues including cultural issues and the inherent value of education as well as increased communication skills and confidence, both clinically and educationally focused, along with personal growth. One respondent stated:

*“Every trip I make helps me grow personally in a variety of different ways, usually in terms of adaptability, team-work, learning how to mentor and support staff and students with varying needs and learning styles, coping with inadequacies in resources and infrastructure”.* UK faculty respondent 11

Another reported:

*“I cannot forget the experience of seeing people struggle for the basics in life and have found some new perspective in my own life in relation to this”.* UK faculty respondent 8

### Overall impact of the visit

The faculty were asked to rate the overall effect of the overseas experience, using a score of 1–10 (1-no impact, 10 maximum impact) and 12 responses were received (Table 1). Lifestyle choices - life work balance (rating 7.83) had the most impact followed by, professional development (rating 7.42), and teaching skills (rating 7.08). Clinical and research skills scored lower ratings (4.83 and 4.20).

**Table 1:** Rating of the effect of the overseas visit

How would you rate the effect of your overseas experience on a scale of improvement from 0 to 10 with 0 being ‘no effect’ and 10 being ‘maximum effect’		
Answer Options	Rating Average	Response Count
Your clinical skills	4.20	10
Your managerial skills	6.00	11
Your professional development	7.42	12
Your teaching skills	7.08	12
Your research skills	4.83	12
Your lifestyle choices – life work balances	7.83	12
Answered question		12
Skipped question		2

## Discussion

The health partnership succeeded in achieving it aims and objectives, with a successful established, successful degree course now in existence. The impact of the course has resulted in the expansion of palliative care services across Sub-Saharan Africa. All the students remain working in palliative care and several have supported the development in services in areas that previously had limited access to palliative care. Interestingly the programme clearly had a positive impact for the individual UK faculty members and also suggested benefits for the wider NHS. Professionally volunteers reported enhanced skills both clinical and educational, and that the experience contributed to their confidence, one respondent reporting that this aided their preparation for taking up a medical consultant post in the NHS. Personally, the opportunity of working in a resource limited country has had a major impact on the volunteers. This personal impact on lifestyle choices and work life balance is clearly worthy of further investigation. Interestingly, several of the UK faculty have continued their contact with the team in Uganda and are seeking to undertake further visits to the Hospice with some involved in another THET funded project to integrate palliative care into health systems in Uganda, Rwanda, Kenya and Zambia.

There are some limitations against the design of the study, which need to be taken into account in the interpretation of the findings. The study employed an electronic survey and although this had an very good response rate, provided a degree of anonymity, met the practical value of collecting data from a sample spread across the UK (and some had returned to Uganda), it did not allow us to probe into their responses. A qualitative study adopting a longitudinal data collection process, would be invaluable to follow up this survey.

The study only included one health partnership and although the findings on the personal value of volunteering resonate with the literature on volunteering in palliative care settings, it may not automatically cross into other clinical specialties. Similarly the value for the respondents on preparing them for becoming a medical consultant and additionally working with less supervision, require more detailed exploration before being formally recognised as an added value to the NHS of supporting health partnerships.

## Conclusions

The World Health Assembly resolution on palliative care passed in May 2014 emphasised both the need for integration of palliative care into existing health systems and the need to train health professionals to provide care across different levels of service provision [25]. Intervention is clearly needed to achieve this goal and further expansion of models of volunteering such as the model reported in this paper are recommended. This model not only contributed to the expansion of the palliative care workforce but had an additional value of positively impacting on the volunteers themselves and their clinical practice.
